# Comparison of ${B}_{1}$^+^ and SAR efficiency for a high-impedance metamaterial shield with different remote RF arrays at 7 T MRI: A simulation study

**DOI:** 10.1007/s10334-025-01295-7

**Published:** 2025-09-11

**Authors:** Ignacio N. López-Martínez, Mark E. Ladd, Rita Schmidt, Stephan Orzada

**Affiliations:** 1 Medical Physics in Radiology, German Cancer Research Center (DKFZ), Heidelberg, Germany; 2 Faculty of Physics, Heidelberg University, Heidelberg, Germany; 3 Department of Brain Sciences, Weizmann Institute of Science, Rehovot, Israel

**Keywords:** Metamaterials, MRI, Ultra-high field, Electromagnetic simulations

## Abstract

**Introduction:**

This study explores high-impedance surface (HIS) metamaterial shields for enhancing the transmit field in whole-body MRI at 7 T. We studied the possibility of placing a metamaterial layer between the gradient coil and bore liner using electromagnetic simulations to evaluate *B*_1_^+^ and SAR efficiency across different impedances.

**Materials and methods:**

Simulations were performed in three stages, first metamaterial design and characterization, then single-element dipole simulations with a homogenous phantom, and finally, simulations including a four-element arrays with a virtual body model, including the whole scanner geometry. Four antenna types were evaluated for *B*_1_^+^ and SAR efficiency.

**Results:**

Due to space constraints the metamaterial does not reach high enough impedance, resulting in minimal performance gains for most antennas. However, fractionated dipole arrays with inductances showed increased SAR efficiency and a larger field of view. Higher impedance values (above 1000 Ω) reduced losses and enabled higher-order wave modes, improving efficiency. Intermediate impedances (10⁻^2^–10^3^ Ω) introduced significant losses, potentially causing heating and detuning.

**Conclusions:**

HIS metamaterials can enhance transmit performance in 7 T MRI but require careful optimization of impedance, material losses, and antenna design. These factors must be considered to ensure both efficacy and safety in ultra-high-field applications.

## Introduction

It is well-established that higher main magnetic fields (${B}_{0}$) can increase the signal-to-noise in magnetic resonance imaging (MRI) in a superlinear manner [1]. Nevertheless, ultra-high field (UHF) MRI (≥ 7 T) faces challenges like transmit field (${B}_{1}^{+}$) inhomogeneities due to the shorter wavelength at increased frequency [2, 3]. At UHF, this wavelength is comparable in size with the human body, and, therefore, destructive interferences can generate regions of low flip angle, so called “dropouts” of signal in the image. Similarly, inhomogeneities in the transmission fields at UHF can cause localized increases in electric fields, leading to “hot spots”, or locations with a relatively high specific absorption rate (SAR) [2].

To address these problems, different arrays of radiofrequency (RF) coils have been tested and developed for UHF MRI, including loop coils, dipoles with different geometries, microstrips, and others [4, 5]. These coils are often shielded by copper to contain the electromagnetic fields inside the area of interest and to prevent interaction with other MR components. These conventional shields can be approximated to impose perfect electric conductor (PEC) boundary conditions. A PEC placed near the antenna is known to cause destructive interference by introducing a phase shift in the incoming wave, due to the generation of opposing mirror currents in the shield, which also leads to amplitude attenuation [6].

A new option to implement shielding and reduce signal dropouts is utilizing metamaterials. Metamaterials are periodic structures engineered to exhibit novel electromagnetic properties that may not occur in nature, leading to a range of applications in manipulating electromagnetic fields. In particular, high-impedance surface metamaterials (HIS) have been of interest due to their phase reflection properties. In MRI, various metamaterial arrays with distinct functionalities have been introduced, including decoupling elements [7], metamaterial absorbers for SAR reduction [8], flexible metamaterials to increase local sensitivity [9, 10], and others [11].

Sievenpiper et al. [6] were first to propose a HIS structure in mushroom-like shape with two layers. The study of the properties of such structures can be simplified by comparing them to a parallel LC circuit. Inductance is generated by the current flowing through the top and bottom layers, while the top layers contribute to capacitance, effectively behaving as an LC circuit.

The impedance of the structure, ${Z}_{\mathrm{HIS}}$, can be described as:

1 $${Z}_{\mathrm{HIS}}=\frac{j\omega L}{1-{\omega }^{2}\mathrm{LC}},$$

where $\omega$ is the resonance frequency given by:

2 $$\omega =\frac{1}{\sqrt{\mathrm{LC}}}.$$

The reflection behavior of a HIS can be studied by the reflection coefficient of the surface, defined as:

3 $$\Gamma =\frac{{Z}_{\mathrm{HIS}}-\eta }{{Z}_{\mathrm{HIS}}+\eta }=\left|\Gamma \right|{e}^{j\Phi },$$

where $\eta$ is the wave impedance of the propagation medium and $\Phi$ is the phase of reflection. For a normally incident wave, the phase of reflection is given by:

4 $$\Phi =\mathrm{Im}\left\{\mathrm{ln}\left(\frac{{Z}_{\mathrm{HIS}}-\eta }{{Z}_{\mathrm{HIS}}+\eta }\right)\right\}={\mathrm{tan}}^{-1}\left(\frac{\mathrm{Im}\left(\Gamma \right)}{\mathrm{Re}\left(\Gamma \right)}\right).$$

At ${Z}_{\mathrm{HIS}}=0$, $\Gamma =-1$ and $\Phi (-1)=\uppi$, while for ${Z}_{\mathrm{HIS}}\to \infty$, $\Gamma =1$ and thus $\Phi \to 0$.

This means that when the wave frequency reaches the resonance frequency $\omega$ of the metamaterial, the impedance increases rapidly, leading to a 0° phase shift of the reflected wave. Therefore, a HIS can be considered a special case of a perfect magnetic conductor (PMC) within a specific frequency range. This boundary condition reflects the wave in a constructive manner when placed near the antenna, ideally increasing the overall ${B}_{1}$^+^ signal inside the patient and increasing the efficiency of the antenna.

The idea of using this reflection property in MRI is not new. Several publications have explored local transmit coils featuring HIS. For example, Issa et al. [12] proposed a shield to increase the $H$-field by 42% in 1.5 T MRI. Saleh et al. [13] showed similar results for 7 T by increasing the $H$-field by 47%. Chen et al. [14] reported slight improvements in penetration depth and homogeneity for local arrays for head MRI at 7 T and increase of 7% of the averaged transverse ${B}_{1}$^+^ field. The same group also tested a local breast coil backed with a HIS and showed an increase in ${B}_{1}$^+^ efficiency and SNR. While these studies have demonstrated promising results for local arrays, limited research has been conducted in the context of whole-body imaging. A recent study by van Leeuwen et al. [15] demonstrated that SAR hot spots in a 3 T birdcage body coil can be mitigated and SAR efficiency improved; however, this comes at the expense of an increased axial extent of the ${B}_{1}$^+^ field, or field of view (FOV), which consequently reduces transmit efficiency.

In this work, we further explore the use of HIS shields using a metamaterial design in whole-body imaging at 7 T MRI to increase the transmit signal by generating constructive interference with the RF shield, with the potential to change the transmit field distribution and, ideally, increase signal in anatomically challenging regions. Our approach was to study the feasibility of an integrated array of antennas placed in the gap between the gradient coil and the patient bore liner. Given the space constraints, the metamaterial was designed considering a maximum thickness of 3 mm. The structure was based on the work of Chen et al. [16], with increased inductance to reduce the thickness while still being able to tune the metamaterial to a working frequency of 297.2 MHz. The study of the metamaterial properties was done using electromagnetic simulations, where the ${B}_{1}^{+}$ efficiency and SAR efficiency were evaluated. Furthermore, high theoretical impedances were tested for different antennas to assess the influence of ${Z}_{\mathrm{HIS}}$ on the field distribution and the transmit efficiency.

## Materials and methods

The project was divided into three main stages: first, the design and characterization of the metamaterial; second, simplified simulations using single elements with different magnitudes of shield impedances in a rectangular phantom; and third, the simulation of four-element antennas in a more realistic simulation including the complete scanner and the realistic body model DUKE [17]. All simulations were performed with CST Studio Suite 2021.

### Design of the metamaterial

**Fig. 1:**
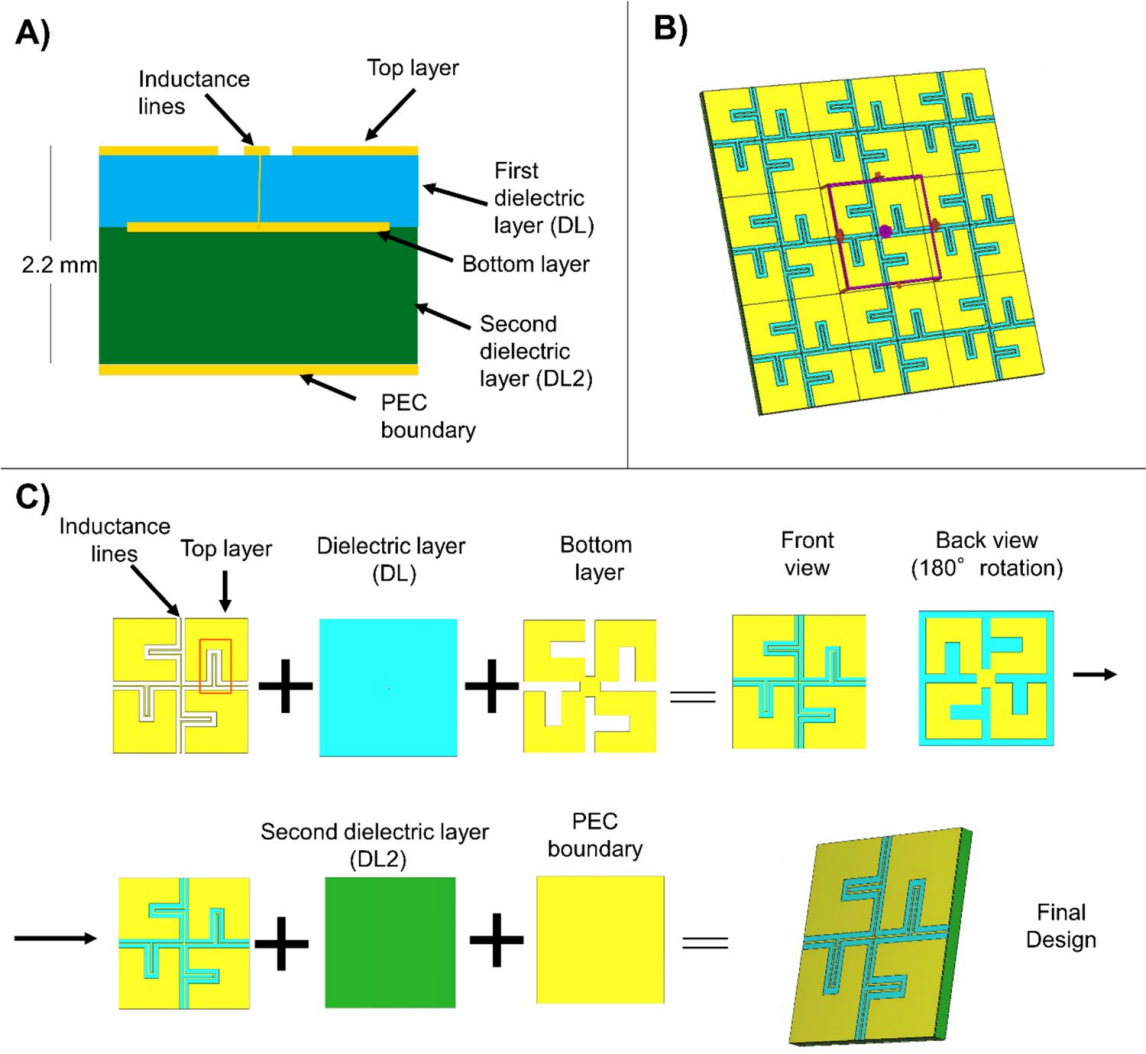
**A** Schematic view of the metamaterial layers in a side view of the structure. **B** Metamaterial structure in a frontal view for a 3 × 3 array of the unit cells. **C** Layer by layer decomposition of the metamaterial, the red rectangle in the first subfigure highlights the meander elements in the inductance lines

The metamaterial design was based on the cross-shaped structure from Chen et al. [16]. It was restructured to minimize its thickness due to space constraints, as the gap between the gradient coil and patient bore liner of our 7 T system is 34 mm. Thus, leaving some distance between the elements, the configuration allows less than 10 mm for the metamaterial and the cables (5.4 mm). To achieve this, the dielectric layers of the metamaterial were moved closer together and the conductor wires were extended by adding meanders, while part of the copper of the top layer reduced. This ensured that the LC ratio of Eq. 2 remained constant at the working frequency of 297.2 MHz while reducing the overall thickness of the metamaterial. The total thickness of the metamaterial was set to 2.2 mm, consisting of 3 copper layers with intervening dielectric material. The top floating layer facilitates easy connection to nearby PCBs, simplifying the manufacturing process. The full structure can be seen in Fig. 1.

Floquet port excitations, which model wave propagation at various incidence angles by introducing phase shifts across the boundaries of a periodic unit cell, were used to represent an infinite array using a single unit cell, significantly reducing simulation complexity. This allows us to tune the structure to the desired resonance frequency (297.2 MHz) by adjusting parameters such as the unit cell size, layer thickness, or other design values listed in Table 1, to achieve high impedance. The influence of material choice on the resulting impedance is discussed in Section. "Discussion and outlook". A co-planar array of these unit cells was used to study current mitigation within the material, a key property of PMC boundary. Additionally, a dispersion diagram was generated using the eigenmode solver in CST to examine how transverse electric (TE) and transverse magnetic (TM) modes propagate. This analysis was restricted to the irreducible Brillouin zone, the smallest segment of the reciprocal space that fully captures all unique wave behavior due to the symmetry of the periodic structure.

**Table 1.**
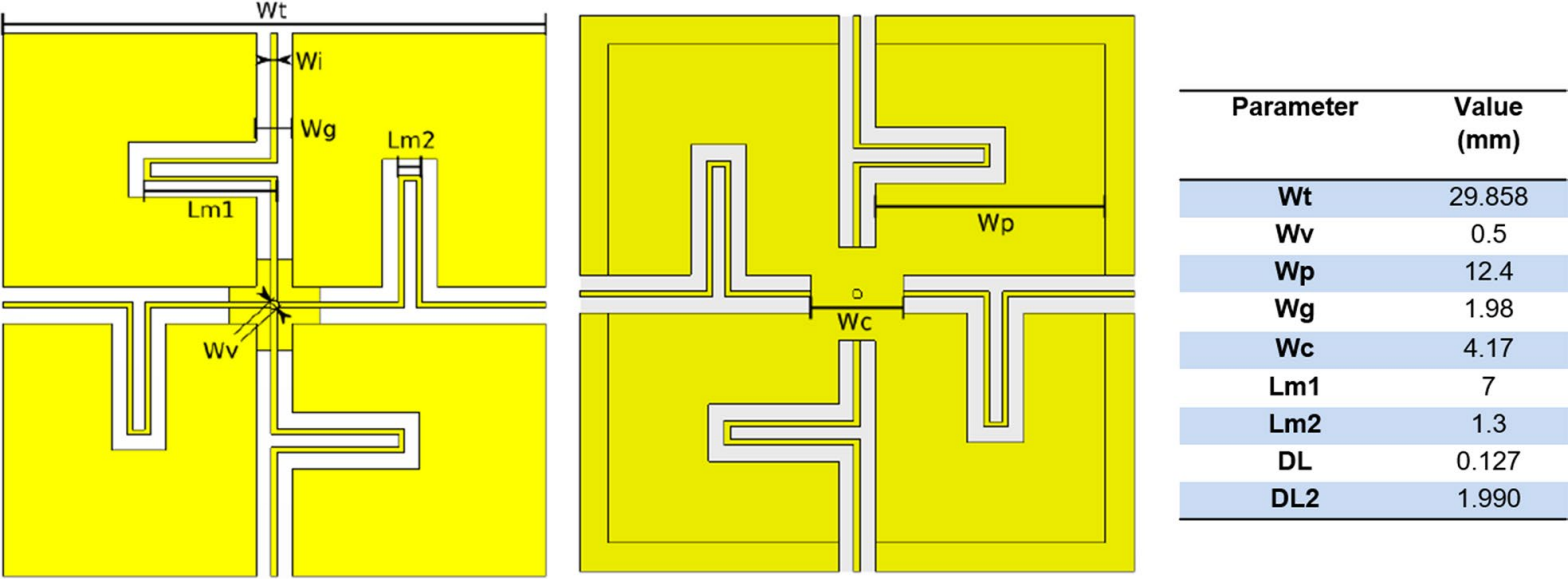
Parameters and values for the metamaterial tuned to working frequency of 297.2 MHz

DL and DL2 are the dielectric layer thickness as shown in Fig. 1A. The left figure represents the frontal view and the central figure the back view of the metamaterial (180° rotation) without the dielectric layers between them.

### Single-element simulations

**Fig. 2:**
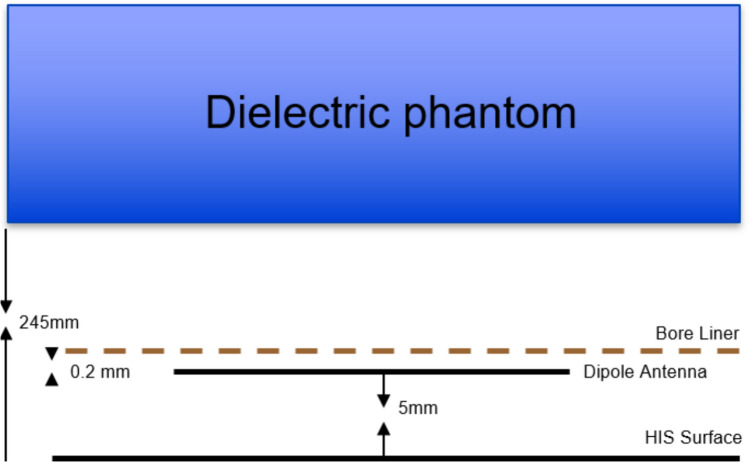
Diagram of single element simulation for remote antenna. The simulations were performed both with and without the bore liner

Simplified electromagnetic simulations (Fig. 2) in a rectangular phantom (500 mm × 500 mm × 230 mm, $\sigma$=0.57 $\frac{{\mathrm{S}}}{{\mathrm{m}}}$, $\epsilon_{r}$=44, ${\mu }_{r}$=1, $\rho$= 1000 $\frac{\mathrm{kg}}{{\mathrm{m}}^{3}}$) using different magnitudes of shield impedance (from ${10}^{-4}$ to ${10}^{3}$) were performed for a half-wave dipole with length of 466 mm and a short dipole with a length of 50 mm. The antennas were normalized to 1W accepted power. Additionally, the simulations were used to study the loading effect of the bore liner on the antennas, and the SAR efficiency and SAR_max_ were obtained.

### Four-element simulations

**Fig. 3:**
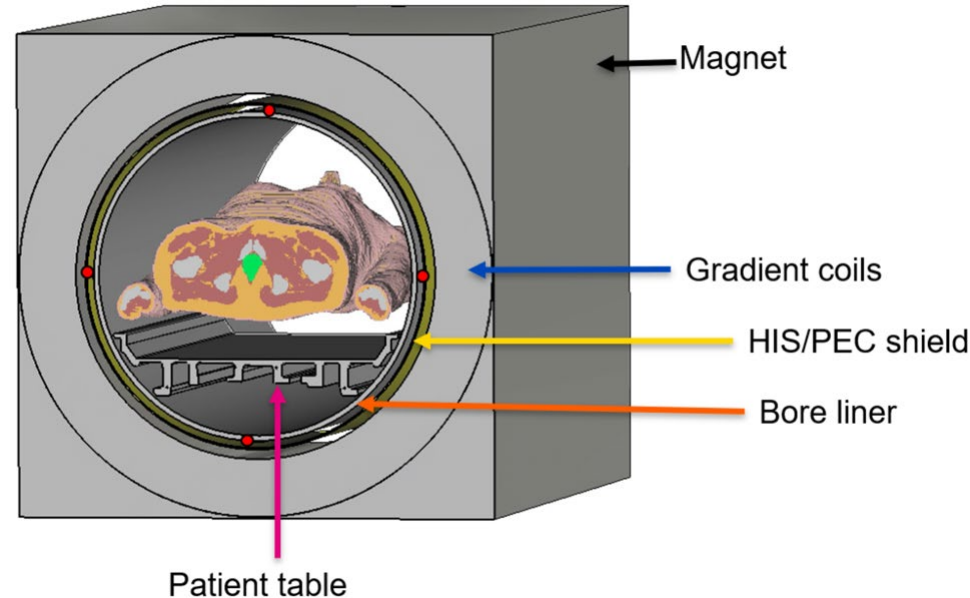
Transversal view of the simulation setup. From the exterior to the interior layer: magnet, gradient coils (Siemens AS095), HIS/PEC shield, antennas (red dots), bore liner, and patient table with patient

More realistic simulations with a four-channel array were performed, including the body model Duke [17] and the entire scanner structure. The scanner design was based on the Siemens Magnetom 7 T with passively shielded magnet and AS095 gradient coils. The bore liner has an inner radius of 59.5 cm, and the total length of the scanner is 3 m. The patient table was also included, as shown in Fig. 3. The metamaterial was added as a cylindrical shield between the bore liner and the gradient coils (simplified as PEC) and simulated as a material with the corresponding impedance.

#### Antennas

Four antenna types were tested for the array, positioned as shown in Fig. 3.

Each antenna was tuned with an LC circuit to minimize input reflection. The tuning ensured that the S-parameters (${S}_{\mathrm{1,1}}$…${S}_{\mathrm{4,4}}$) were at least -15 dB when the elements were placed within either a PEC or HIS shield and normalized to 1W stimulated power.

- Half-wave dipole

Dipoles are well known for their uniform RF distribution [18], ease of integration into multichannel arrays [19], and enhanced deep tissue penetration [18], making them a good standard. A 466 mm dipole antenna with a central excitation gap of 20 mm was used. The length was chosen so that the antenna’s maximum field was centered within the geometry when tuned without metamaterial.

- Short dipole

A smaller version of the half-wave dipole was used to study whether the metamaterial could increase the field of view and the possibility of using smaller antennas. The total length was reduced by one-third to 310.6 mm. Smaller versions, like the one used in Section. "Single-element simulations", were found to be heavily loaded by the bore liner and were therefore unsuitable for the study (see "Results").

- Fractionated dipole

**Fig. 4:**
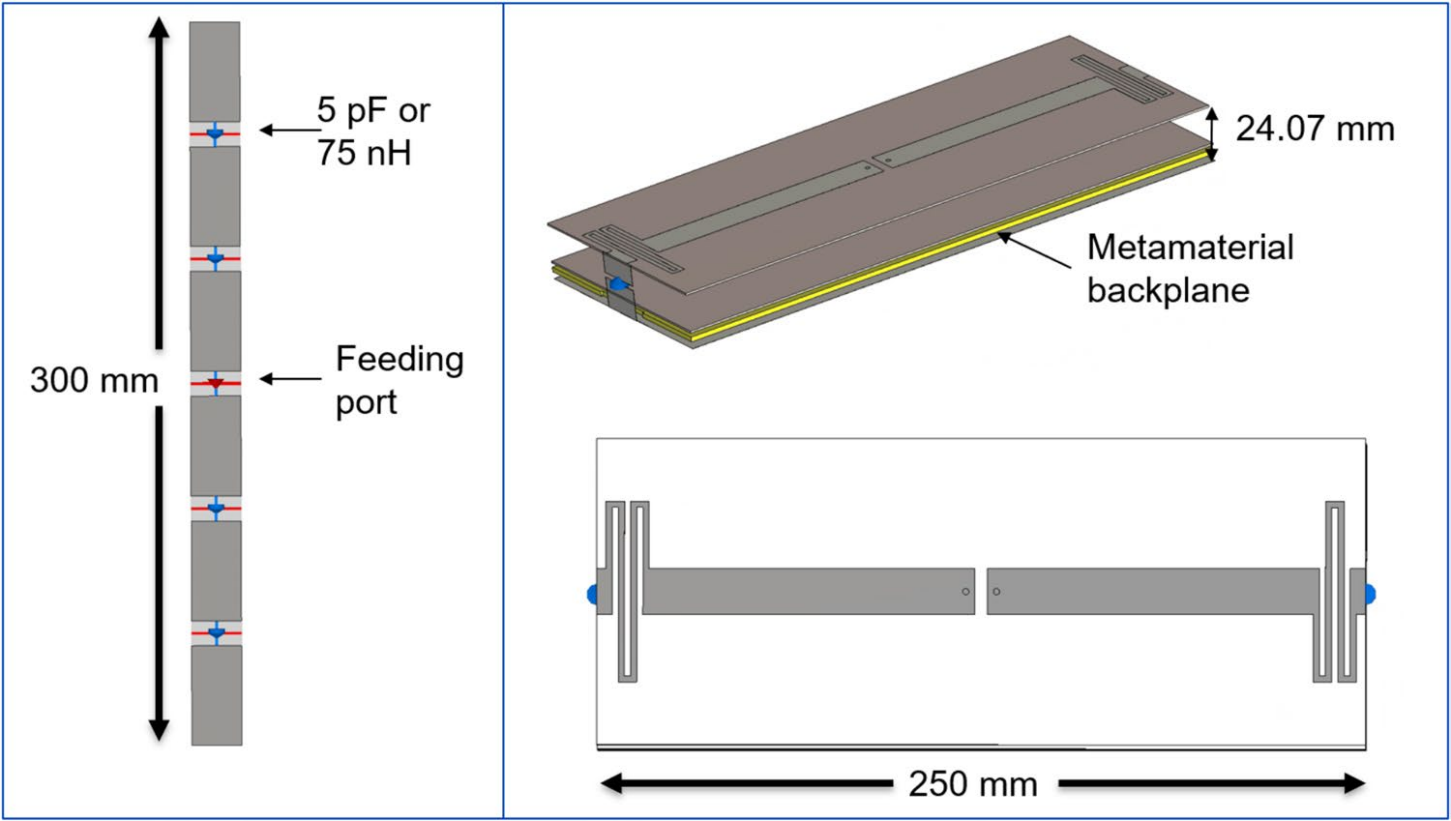
Left: Fractionated Dipole. Red ports indicate feeding points and blue points lumped elements. Right: Meander stripline backed with the metamaterial shield. Central feeding was achieved by two central vias (circles in the center of the antenna)

Raaijamakers et al. [20] showed that using a fractionated dipole with four lumped elements and central feeding (Fig. 4, left) could improve SAR efficiency compared to plain dipoles. To investigate this, two cases proposed in their paper were studies: an array with lumped elements of 5 pF and another with lumped elements of 75 nH.

Adding capacitors to the antenna is similar to decreasing the effective length of the antenna, therefore, leading to a more localized current distribution in the center of the antenna as it is expected from a short dipole. However, ${B}_{1}^{+}$ at depth remains unchanged in comparison to a plain dipole of the same size making it a better alternative than a short dipole [20]. In contrast, inductors increase the effective length and can make the antenna support up to two wavelengths.

Both cases (5 pF and 75 nH) were studied by Raaijamakers et al. [20]. 5 pF was chosen because their simulated results indicated an increase in ${B}_{1}$^+^ without a large compromise in SAR efficiency, while 75 nH was selected because it was the inductance that they empirically tested.

- Meander stripline

Modern whole-body imaging techniques such as RF shimming, SENSE [21, 22], and TIAMO [23] require multiple RF coils. As a result, coupling between the elements becomes an important parameter. Therefore, elements like the microstrip line with meander elements investigated by Rietsch et al. [24] are an interesting approach to reduce coupling due to the extended electrical length of the microstrip line thanks to the meander elements at the end of the structure (Fig. 4, right). This type of element is centrally fed with two ports and contains lumped elements at the two ends connected to the PEC backplane behind the metamaterial.

In contrast to the other elements, the metamaterial was not added as a cylindrical shield around the meanders due to space constraints caused by the dielectric layers of the antenna, where the curvature of the cylinder would interfere with the antenna structure, but instead a smaller plate was used to fit in the geometry of the scanner as well as minimizing the necessary space as shown in Fig. 4.

## Results

### Metamaterial design

The metamaterial was tuned to a resonance frequency of 297.2 MHz, with the final parameters described in Table 1. The total thickness of the material is approximately 2.2 mm considering a copper thickness of 0.3 mm, achieving a smaller thickness than the starting design (3.4 mm) in Ref. [16]. The impedance depends on the losses of the material. Realistic losses for the conductors and RT6006 as a dielectric material with relative permittivity constant ${\varepsilon }_{r}=6.45$ were used. Under these assumptions, the metamaterial has an impedance magnitude near 300 Ω (309 + i48 Ω) at resonance frequency. 300 Ω was used as a conservative estimate due to possible errors in fabrications and the infinite array approximation. While the impedance can be studied in terms of their complex and real values, Chen et al. [14] showed that the difference in the field distribution (H and E fields) is not significant in the ranges of Re(${Z}_{\mathrm{HIS}}$) >  = Im(${Z}_{\mathrm{HIS}}$), specially for larger impedances where the difference becomes negligible even for|${Z}_{\mathrm{HIS}}$|= Im(${Z}_{\mathrm{HIS}}$).

**Fig. 5:**
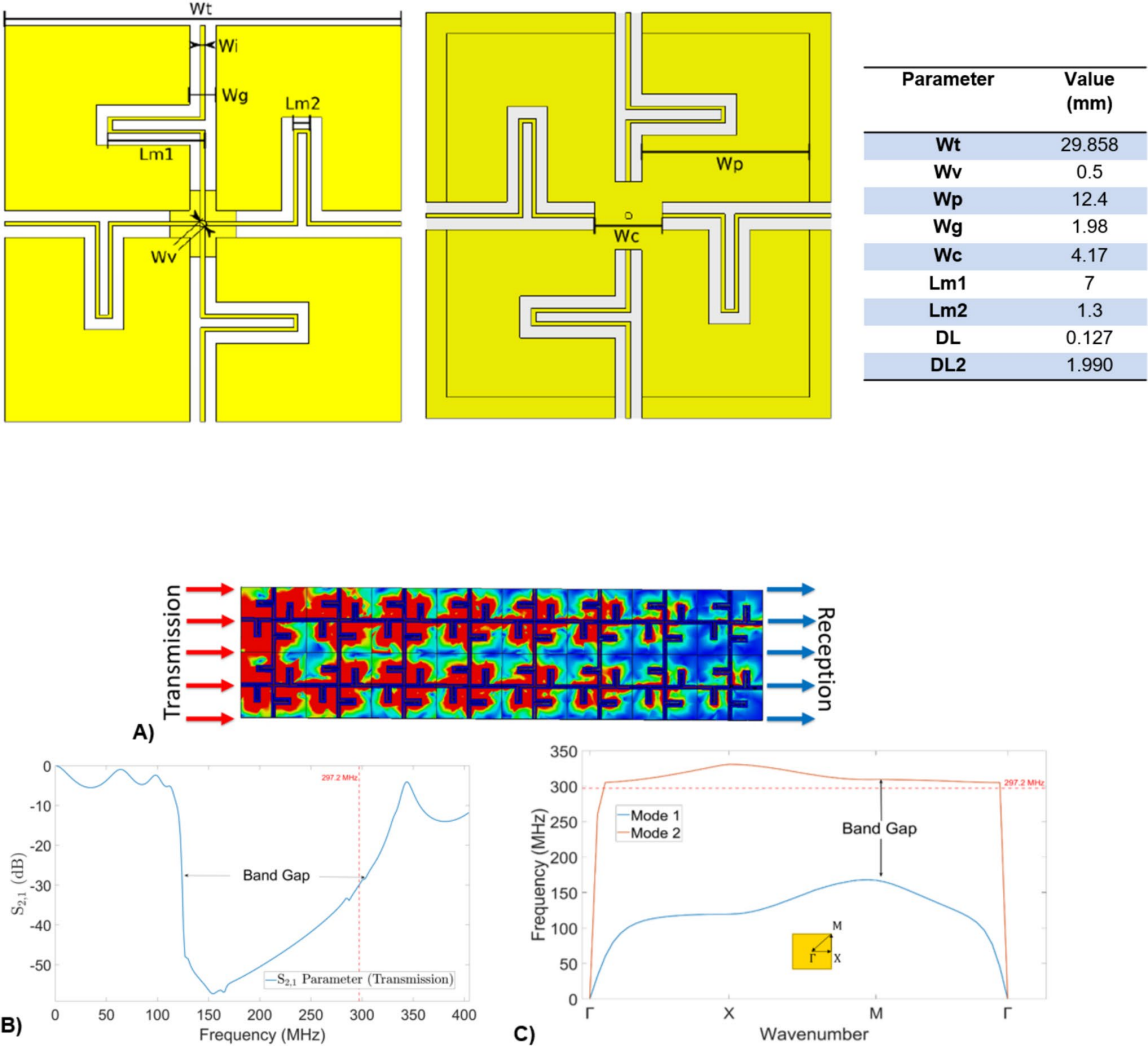
**A** Simulation of surface current density in a co-planar array of unit cells (2 × 8) for 297.2 MHz. Red represents a higher current density. **B** S2,1 parameter (Transmission) of the metamaterial for the co-planar simulation in **A**. **C** Dispersion diagram for TM-like (Mode 1) and TE-like (Mode 2) modes

The co-planar transmission line model (Fig. 5A) shows a band-gap behavior in transmission (${S}_{\mathrm{2,1}}$< − 20 dB) for frequencies ranging from 124 to 320 MHz (Fig. 5B).

The dispersion diagram of the irreducible Brillouin zone is shown in Fig. 5C. The purpose of the simulation is to determine the eigenfrequencies of the material along the triangular path $\Gamma -{\rm X}-{\rm M}-\Gamma$. Any other frequency with the same direction (wavenumber) but different frequency will become an evanescent wave, therefore decaying exponentially rather than oscillating in the material. The results show a bandgap in the dispersion diagram that aligns with the bandgap observed in the transmission line model. The advantage of this approach is the ability to study wave propagation in multiple directions rather than being limited to a single transmission path.

### Single-element simulations

**Fig. 6:**
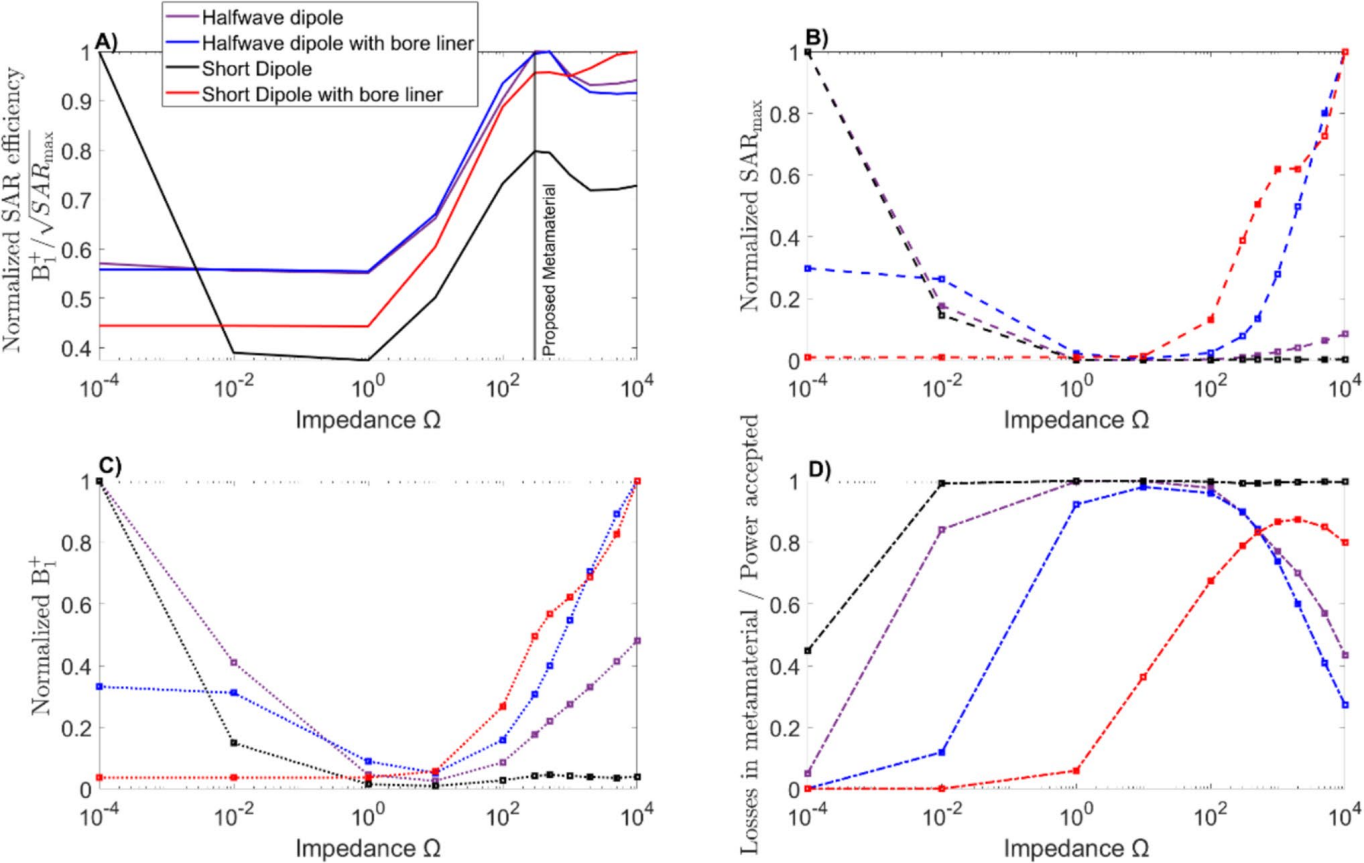
Single element simulations. **A** Average SAR efficiency (${B}_{1}$^+^/$\sqrt{{\mathrm{SAR}}_{\mathrm{max}}}$) inside a rectangular phantom (500 mm × 500 mm × 230 mm) ($\sigma$=0.57 $\frac{{\mathrm{S}}}{{\mathrm{m}}}$, $\epsilon_{r}$=44, ${\mu }_{r}$=1 $\rho$= 1000 $\frac{\mathrm{Kg}}{{\mathrm{m}}^{3}}$). **B** ${\mathrm{SAR}}_{\mathrm{max}}$ inside the phantom for different magnitudes of shield impedance for a single element normalized to the maximum of each antenna. **C** Normalized sum of ${B}_{1}$^+^ inside the phantom to the maximum of each antenna. **D** Metal losses in the metamaterial over the accepted power for different impedances. The maximum (black line) is 0.99

For single-element simulations, a simplified geometry was considered (Fig. 2). The SAR efficiency and ${\mathrm{SAR}}_{\mathrm{max}}$ were analyzed. Figure 6A illustrates the average SAR efficiency inside the entire phantom. In general, it can be seen that at the metamaterial impedance the SAR efficiency increases for all antennas, which correlates with the results of Chen et al. [14] where the PMC distribution was wider, thereby increasing the FOV and the overall ${B}_{1}$^+^ inside the phantom.

However, this increased SAR efficiency is not product of an increment in ${B}_{1}$^+^. This effect is caused by a sharp decrease in the power delivered to the phantom due to increased losses in the metamaterial, thus highly reducing ${\mathrm{SAR}}_{\mathrm{max}}$ (Fig. 6B) and the overall ${B}_{1}$^+^ inside the patient (Fig. 6C). When the impedance goes beyond certain threshold (that depends on the antenna), the losses starts to decrease, and the ratio between ${\mathrm{SAR}}_{\mathrm{max}}$ and ${B}_{1}$^+^ start to make the antenna more SAR efficient, but at the cost of input power.

It was observed during tuning that the antenna’s bandwidth rapidly increased in the range of 0.01–100 Ω, and above 300 Ω the bandwidth began to decrease for half-wave antennas. This behavior is related to the losses associated with the Q-factor (i.e., higher bandwidth results in higher losses). As a result, the power deposited in the shield was analyzed and it was found that most of the power was being lost in the metamaterial and that the behavior of this loss is dependent on the antenna design and the presence of the bore liner.

Additionally, the short dipole simulation revealed significant loading effects when placed near the bore liner, making it considerably less efficient with a PEC shield. Therefore, it is not suitable for comparison and its small FOV further limits its realistic applicability in whole-body imaging.

### Four-element simulations

**Fig. 7:**
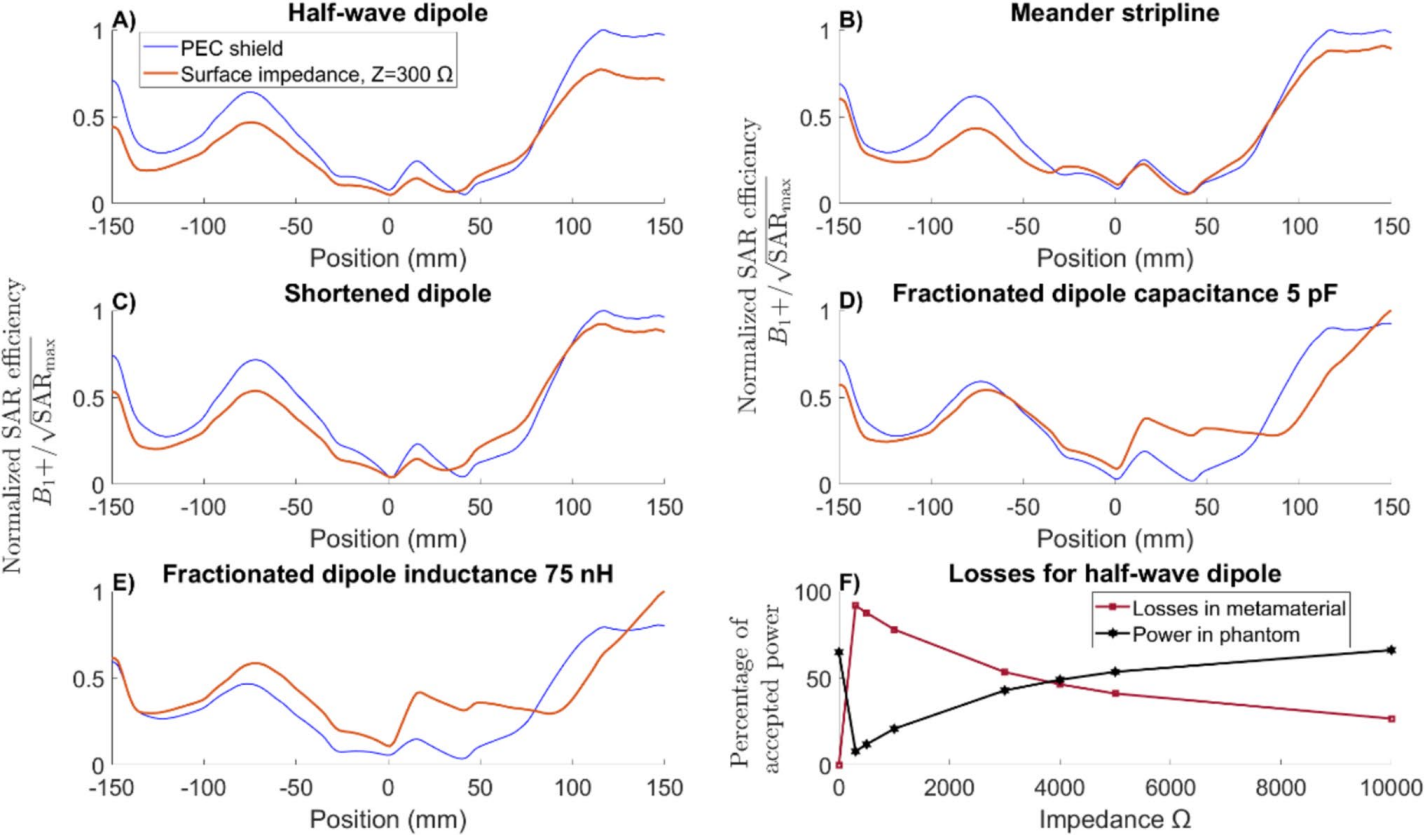
Four-channel simulation. **A**–**E** Normalized SAR efficiency (${B}_{1}$^+^/$\sqrt{{\mathrm{SAR}}_{\mathrm{max}}}$) in the central slice along the left–right direction (arm to arm) inside the Duke phantom for the 4-channel configuration for different antenna elements. **F** Losses in the metamaterial and power deposited into the phantom as a function of the magnitude of shield impedance. Efficiency is improved for the HIS (red line) compared to a PEC shield (blue line) only for the fractionated dipole

The four-channel simulations, which included the entire scanner setup (Fig. 3), showed that at ${Z}_{\mathrm{HIS}}=300$ Ω there was no significant gain in efficiency at the central slice of the patient compared with a PEC shield, except at specific depths for fractionated dipoles (Fig. 7A–E).

To further investigate this, we studied the power losses in the materials as a function of metamaterial impedance. Compared with a traditional PEC shield, the majority of the input power is lost within the metamaterial itself when HIS are used (Fig. 7F). This of course decreases the SAR but also reduces the ${B}_{1}$^+^ field inside the patient, making it require more power to achieve the same signal (From 1.6 times to more than 10 times depending on the impedance).

**Fig. 8:**
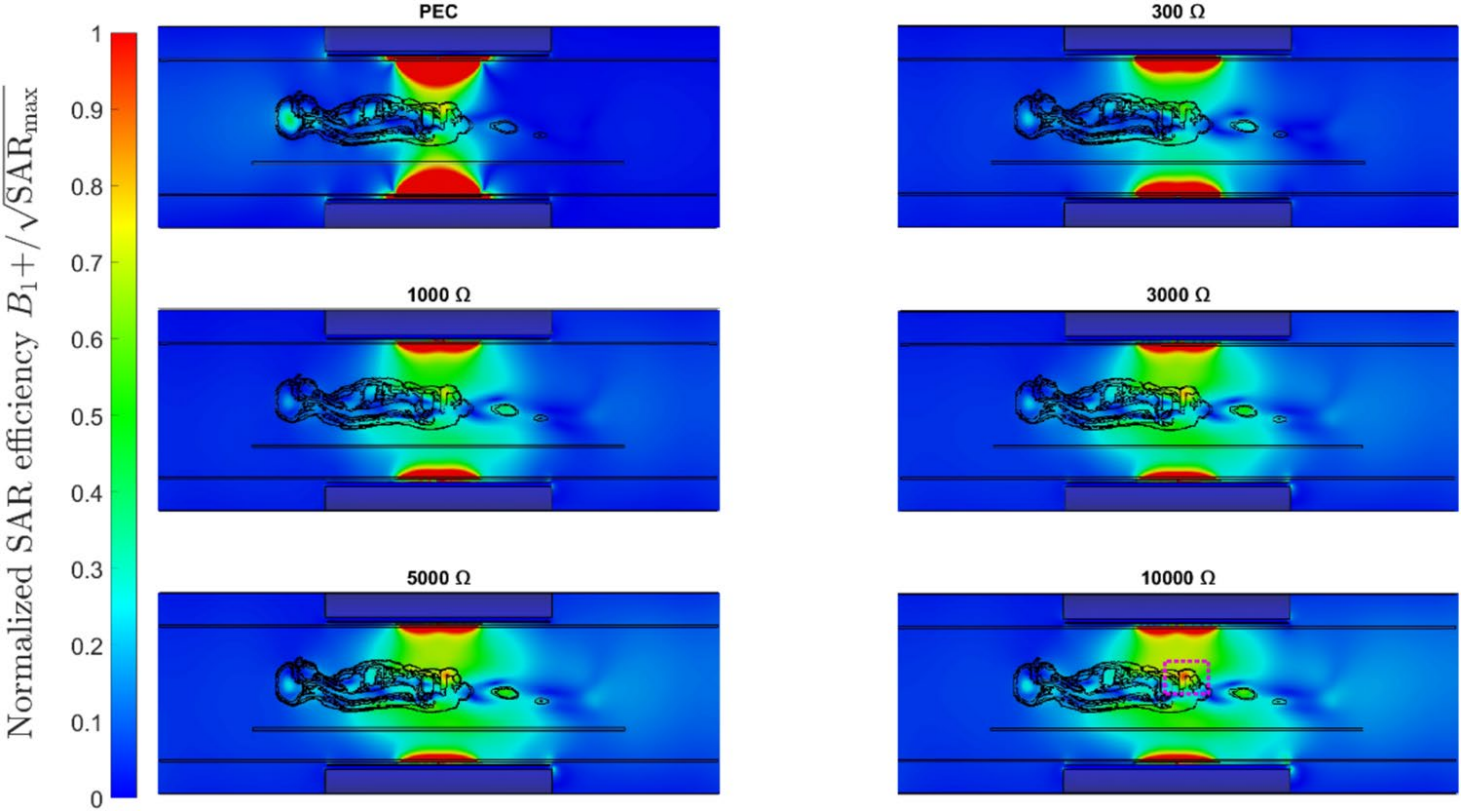
Normalized SAR efficiency (${B}_{1}$^+^/$\sqrt{{\mathrm{SAR}}_{\mathrm{max}}}$) in the sagittal plane for the 4-channel configuration of the half-wave dipole for different magnitude of impedances. The magnet, gradient coils, patient table, and bore liner are included in the simulation domain

Although the 2D distribution of SAR efficiency (Fig. 8) does not show a clear improvement at the metamaterial impedance, it is noteworthy to notice that as the impedance further increases there is a wider distribution and higher peak at the center of the patient (pink square Fig. 8) for half-wave dipole antennas. This type of distribution could potentially cover a larger FOV with high B_1_^+^ intensity, making whole-body imaging at UHF more feasible.

## Discussion and outlook

State-of-the-art RF coils perform extremely well regarding homogeneity and sensitivity in low fields. However, when the ${B}_{0}$ field causes the wavelength to be on the same order as the body size, the distortions for whole-body imaging can be significant. PEC shields may not be ideal, making HIS appear to be an interesting option. In this work we designed and analyzed a HIS metamaterial structure; however, our results indicate that it did not achieve a sufficiently high enough impedance to improve the efficiency of the antenna, except for fractionated dipole arrays. Nevertheless, there is still potential to achieve a larger field of view and greater signal inside the patient at higher impedances (Fig. 8). A straightforward approach to increase the metamaterial impedance would be to increase its thickness by increasing DL2 in Fig. 1, for example, adding 10 mm of substrate would increase the impedance to 700 Ω, but further increment would not increase the impedance, due to the intrinsic loses in the dielectrics. Using dielectrics with lower losses can increase the reachable impedance. For example, using RO4360G2 as a dielectric material with higher losses compared to RT6006 effectively would reduce the metamaterial impedance by 13%, while using a lossless dielectric material would increase the impedance to around 515 Ω.

The main problem of “intermediate” impedances (on the order of 0.01–1000 Ω) are losses generated by the metamaterial. As shown in Fig. 7F, the losses become comparable with the input power, requiring increased power to get the same B_1_^+^ inside the patient. This in itself is not a hardware limitation, as RF antennas can tolerate several times the typical operational input power without problems. However, it raises the question of whether the absorbed power in the material can eventually lead to detuning by heating, altering the properties of the antenna and potentially putting the patient at risk.

In general, our results show relatively good agreement with previous studies demonstrating that HIS can expand the FOV of antennas [14, 15]. However, this work provides a more in-depth analysis of losses and tuning for different shielding impedances. We conclude that the losses should not be overlooked, making it crucial to report additional metrics beyond SAR efficiency or H/E maps when evaluating metamaterials in MRI applications.

This study has some limitations that could be addressed in future research. One key limitation is the simplification of the simulated shield using a constant impedance. A more realistic approach would involve simulating an array of unit cells to study possible detuning of the material by specific geometries of the metamaterial. However, this approach is highly computationally demanding due to the large number of mesh cells required to correctly replicate the unit cell properties; nevertheless, our approach was successfully used in previous studies to characterize metamaterial properties [25, 26]. Another consideration is that in this study we presented four types of antennas, where only with one (the fractionated dipole with inductance (Fig. 7E)) the metamaterial showed significant increase in efficiency in the central slice. Other antenna designs may benefit more from the metamaterial, raising the question of whether a specific antenna, array configuration, or shim mode could utilize the metamaterial performance even at 300 Ω.

Figure 8 shows how the ${B}_{1}$^+^ field distribution changes as a function of impedance. Above 1000 Ω the distribution shape begins to converge and the overall efficiency starts to increase inside the patient, resulting in localized increase in SAR efficiency in the central slice of the patient and a larger FOV. Additionally, due to the boundary conditions imposed by the metamaterial, the wave propagation within the antenna happens in a higher-order mode. This may explain the improvement generated in the fractionated dipole antenna (Fig. 7E), because the increased effective length introduced by the meanders can support the complete wavelengths in higher-order propagation.

To conclude, we presented a thin high-impedance metamaterial for 7 T MRI remote arrays and analyzed the overall effect of impedance on different types of antennas in both single-element and four-element arrays. Our findings show that the HIS metamaterial increased the SAR efficiency and FOV when implemented with fractionated dipole antennas, while it had no significant effect on overall efficiency in other configurations within the size constraints used here. This study highlights that a variety of antenna designs should be considered when using high-impedance materials due to the different loading effects they may have on the antenna. Additionally, we found that losses in the material decrease as impedance increases above roughly 1000 Ω, which we conclude is the main limitation for their implementation in the range of 0.01–1000 Ω. However, beyond this range, the overall efficiency inside the patient improves, making it an interesting alternative for whole-body imaging. Overall, the study emphasizes the importance of considering interactions of the investigated antenna types with high-impedance materials to optimize performance.

## Data Availability

The data that support the findings of this study are available on request from the corresponding author IL.
